# A review of environmental and occupational exposure to xylene and its health concerns

**DOI:** 10.17179/excli2015-623

**Published:** 2015-11-23

**Authors:** Kamal Niaz, Haji Bahadar, Faheem Maqbool, Mohammad Abdollahi

**Affiliations:** 1 Pharmaceutical Sciences Research Center, Tehran University of Medical Sciences, International Campus, Tehran, Iran; 2 Department of Toxicology and Pharmacology, Faculty of Pharmacy, Tehran University of Medical Sciences, Tehran 1417614411, Iran

**Keywords:** xylene, health hazards of xylene, genotoxicity, hepatotoxicity, neurotoxicity, review

## Abstract

Xylene is a cyclic hydrocarbon, and an environmental pollutant. It is also used in dyes, paints, polishes, medical technology and different industries as a solvent. Xylene easily vaporizes and divides by sunlight into other harmless chemicals. The aim of the present review is to collect the evidence of the xylene toxicity, related to non-cancerous health hazards, as well as to provide possible effective measurement to minimize its risk ratio. For current study a bibliographic search of more than 250 peer-reviewed papers in scientific data including PubMed, and Google Scholar about xylene was done. But approximately 130 peer-reviewed papers relevant to xylene were included (Figure 1(Fig. 1)). All scientific data was reviewed with key words of “xylene toxicity”, “xylene toxic health effects”, “environmental volatile organic compounds”, “human exposure to xylene”, “xylene poisoning in laboratory workers”, “effects of xylene along with other hydrocarbons”, “neurotoxicity of selected hydrocarbons”, and “toxic effects of particular xylene isomers in animals”. According to these studies, xylene is released into the atmosphere as fugitive emissions from petrochemical industries, fire, cigarette, from different vehicles. Short term exposure to mixed xylene or their individual isomers result in irritation of the nose, eyes and throat subsequently leading toward neurological, gastrointestinal and reproductive harmful effects. In addition long term exposure to xylene may cause hazardous effects on respiratory system, central nervous system, cardiovascular system, and renal system. The health concerns of xylene are well documented in animals and human. It is important to improve health policies, launch xylene related health and toxicity awareness campaigns, to get rid of its dangerous outcomes. Chronic diseases have become a threat to human globally, with special prominence in regions, where xylene is used with other chemicals (benzene, toluene etc.) especially in petroleum and rubber industry. The mechanism of toxicity and interactions with endocrine system should be followed up which is the main threat to human health.

## Abbreviations

TWA = time-weighted average; p = para; m = meta; o = ortho; Bd wt = body weight; d =day (s); DNA = deoxy ribonucleic acid; CYP = cytochrome P; GABA = gamma-aminobutyric acid; Gd = gestational day; SGPT = serum glutamic pyruvic transaminase; x = time (s); mo = month (s); yr = year (s); mg = milligram; kg = kilogram; CNS = central nervous system; GIT = gastrointestinal tract; RER; rough endoplasmic reticulum, RBC = red blood cells; WBC = white blood cells, ppbv = parts per billion volume, EPA = Environmental Protection Agency, NPL = National Priorities List, VOC = volatile organic compound, MDA = malondialdehyde, SOD = superoxide dismutase, GSH-Px = glutathione peroxidase, VOCs = volatile organic compounds

## Introduction

Xylene, or dimethyl benzene C_6_H_4_ (CH_3_)_2_ is an organic aromatic hydrocarbon. It is widely used for the production of ethyl benzene, polyester, and plastic, and also acts as a solvent in paints or varnishes. The individual isomers of xylene are: *ortho*-, *meta*-, and *para*-xylene (Fay et al., 1995[31]). 

### Human exposure to Xylene alone and in conjugation with VOCs

Human life is surrounded by a wide range of volatile organic compounds (VOCs), including xylene causing harmful health effects. Contact to xylene occurs through breath, eye, oral, and dermal route. Health authorities in most countries, like in United States suggest a typical level of xylene as100 ppm in industrial place where xylene is commonly used or produced, and 14 ppm in the usual atmosphere (Langman, 1994[55]). The major sources of xylenes release are petroleum refineries, automobile exhausts. After releasing into the environment xylene may be leached into ground water and enter the human food chain. Human exposure to xylene mostly takes place from working areas where either xylene is used or emitted to the environment.

**Figure 2:**
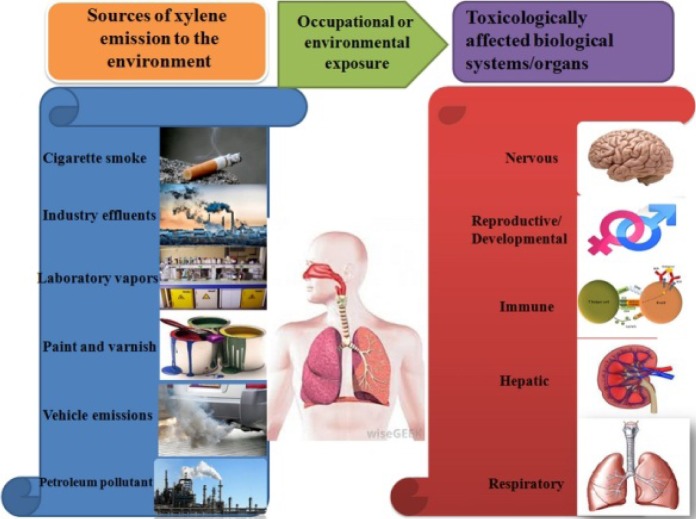
Routes of xylene liberate to the environment and its toxic effects on human health from perspective of biological systems

Xylene is mainly metabolized in the liver via oxidation of methyl groups, followed by conjugation with glycine to yield hippuric acid (Ogata et al., 1970[76]; Šedivec and Flek, 1976[101]), which is excreted through urine. But high amount and dose of xylene harm the liver and even its metabolites damage the hepatocyte also. Some amount of xylene is eliminated via exhalation. The kind and intensity of health effects caused by xylene are determined by different parameters, like exposure route, duration and also individual responds differently to various levels of exposure. Occupations which lead to human xylene exposure mainly include histopathology laboratories, petrochemicals and steel manufacturing, leather and rubber industries shown in Figure 2(Fig. 2) (Fay et al., 1995[31]).

Exposure to mixed benzene, which contain xylene and toluene at low concentration of long term exposure may lead to enhance the content of malondialdehyde (MDA) and action of superoxide dismutase (SOD), and to reduce the action of glutathione peroxidase (GSH-Px) (Tang and Xu, 2005[107]). A study was conducted in the Athens, Greece by Alexopoulos et al. (2006[3]) that the people working or time-out at the city center is the major contribution to xylene and toluene among non-smoking populations. The polluted inhaled air like a painting and mode of heating in the residential area and mean transportation also contributes to the exposure levels.

VOCs containing xylene and formaldehyde may also impair the memory and hearing ability in mice (Zhang and Song, 2005[130]). Female workers’ working in the jewel processing are more prone to DNA damage in the peripheral blood cells that are exposed to xylene, benzene and toluene simultaneously (Huang et al., 2010[42]). There were fatigue, conjunctivitis, deterioration in memory and hand degreasing. Low level exposure to mixed xylene containing benzene and toluene may lead to incoordination, memory loss, coryza, catamenai disruption, dermatitis and pharyngitis. So these mixtures are harmful for reproductive system, nerve system mucous membrane and cuffs (Wang et al., 2004[125]). So it is concluded that these adverse effects having the contribution of xylene exposure also. Xylene has significant noxious effects on the ovarian tissue of mice, which increase the contents of MDA with higher dose, while decreased SOD action as compared to control group. Pathological changes in the ovarian tissue, ovary atrophy and black grains in the middle of follicle with high dose were also observed (Wei et al., 2011[126]).

### Potential environmental levels of Xylene

Xylene has been listed in the National Priorities List (NPL) on the EPA, which is recognized in at least 840 of the 1,684 toxic waste sites (HazDat, 2006[37]). Xylene are freely expatriate to the environment primarily as an elusive emanation from different industries like petroleum refineries, chemical plants, vehicle exhaust and also through volatilization from their use as solvent. This discharge of xylene mainly comes into watercourses and spills on land-dwelling from their usage, stowage and transport of petroleum, and waste products. When xylene is discharged to soil and water, it will ultimately vaporize into the environment. Though, on the basis of flexibility of these constituents in the land, xylene may also percolate into groundwater, where it may persevere for the months (Merian and Zander, 1982[59]).

As almost all chemical substances degrade in the environment. Base on the studies carried out in the U.S. xylene was found < 5 % in water samples. Though, xylene concentration was much higher as 10,000 ppb in contaminated groundwater. There are little data available regarding the concentration of xylene in ground water and soil. Xylene was present < 6 % in the drinking water collected during U.S. drinking water surveys. As single xylene isomers are highly used in industrial plants, individuals who work or live near these settings may obtain huge quantities of individual isomers associated to other isomers. However, xylenes are used in mixed form in gasoline and in other commercial products like paints, so people are generally exposed to a mixture of xylene, and not to single xylene isomers (Merian and Zander, 1982[59]). 

In the overall population the xylene and its metabolites have been identified in the human urine, blood and exhaled air. Xylene exposure is thought to occur via breathing of indoor and workplace air, inhalation of vehicle elusion, drinking of polluted water, smoking and skin absorption of solvents having xylene. A study conducted in the lower Passaic River in New Jersey, xylene was identified in only 4 out of 30 sediment samples was 15, 7, 6, and 5 µg kg^-1^ (ppb) (Iannuzzi et al., 1997[43]). 

In 2004, from 2,794,58,67 and 39 local industrial and processing facilities, the predictable releases of *p-*xylene, *o-*xylene, *m-*xylene, and mixed xylene to the environment were 1.4 million pounds, 0.6 million pounds, 0.5 million pounds, 34.8 million pounds respectively. This release accounts for < 0.1 %, 1.4, 4.3, and < 0.1 % of the estimated total atmosphere releases of *p-*xylene, *o-*xylene, *m-*xylene and mixed xylene respectively (Tri04, 2006[75]).

A study was conducted in the landfill gas of the Fresh Kills Municipal Solid-waste Landfill in New York City that the emitted mean concentration of *o-*xylene and *p-/m-*xylene were 2.17 and 5.97 parts per billion volumes (ppbv) respectively (Eklund et al., 1998[24]). Eitzer (1995[23]) identified VOCs released at 08 municipal solid waste composting lavatories. The estimated concentration was different in background air, indoor air, digester air, fresh compost air, and tipping air. While Schauer et al. (2001[99]) stated that release of *p-/m-*xylene and *o-*xylene during pine timber burning were 60.0 and 18.1 mg kg^-1^ of wood burned, respectively. The mean concentration of *p-/m-*xylene in the indoor air of 04 constructed houses and 07 site built houses in the U.S were 0.5-2.7 ppb and 1.4-11.5 ppb, respectively (Hodgson et al., 2000[39]). 

Odermatt (1994[74]) measured the mean concentration of xylene in 03 groundwater monitoring wells at a Shell Oil service station in California were 0.39, 1.23, and 19.35 mg L^-1^. While xylene concentration was 2.8 ppm in the water at oil spill site in northern Virginia (Mushrush et al., 1994[69]). Xylene emissions from four-stroke outward and two-stroke outward machine range from 0.07-0.09 g kW^-1^ and 0.27-0.86 g kW^-1^ respectively (Gabele and Pyle, 2000[33]). So xylene emission occurs in the atmosphere, but exposure having very limited potential health effects because its concentration is lower than corresponding criteria. While in some industrial plants and petroleum refineries processing xylene exposure occurred mixed with benzene and toluene. But in some areas of U.S. continuous exposure of xylene may lead to low potential or non-observable potential health effects.

## Evidence of non-cancerous health effects of xylene

### Hematological effects

Lowengart et al. (1987[57]) reported that children born to parents working in industries having xylene are at high risk for leukemia, although the female had usually normal blood parameters, like WBC (white blood cells), RBC (red blood cells) and platelets once exposed to *p-*xylene for 1-7.5 hours day^-1^ for 5 days. The leukocytosis, high level of serum glutamatic oxaloacetic transaminase (SGOT) and increased alkaline phosphatase were seen in two workers acutely exposed to xylene (Morley et al., 1970[63]). Prolonged exposure to xylene caused a decrease in mean corpuscular hemoglobin, dropped total leukocytes because of decrease in different cells counts due to enhancement of monocytes and reticulocytes (Moszczynsky and Lisiewicz, 1983[68]; Hipolito, 1980[38]). A variety of effects were observed in chronic exposure to xylene via inhalation and oral routes (Condle et al., 1988[17]). However, in all cases solvent mixtures assumed that it constitute benzene, which is also a known chemical lead to cancer of the blood (Bahadar et al., 2014[11]). Therefore, these effects are not solely associated with xylene. Exposure to xylene at different concentration for 10 consecutive days lead to a decrease in weight of the spleen, while mixed xylene at 1,500 mg kg^-1^ day^-1^ for 3 months increased 11 % weight of the spleen, mild polycythemia, and leukocytosis (Condle et al., 1988[17]). Xylene induces leukocytosis by an absolute increase in neutrophil numbers, while it causes reduction in hemoglobin, and erythrocyte counts, however, platelet counts keep high (d'Azevedo et al., 1996[19]). Langman et al. (1994[55]) had described that chronic exposure to *o-*xylene has been associated with thrombocytopenia, leukopenia, and anemia. Occupational exposure to xylene significantly diminishes lymphocytes in the peripheral blood containing intact N-acetyl-beta-D-glucosaminidase (NAG-positive) lysosomes (Moszczynski and Lisiewicz, 1985[67]).

Xylene and ethanol, lead to a diminish in erythrocyte sedimentation rate (ESR), erythrocyte packing difference, presence of macrocytes of blood, and a decreased fluid of erythrocyte membrane in the central region of the phospholipid bilayer in rat (Wrońska et al., 1991[128]). There have been illustrious statistically significant effects in changing differential WBCs and RBCs in animals exposed to single or a mixture of toluene and *m*-xylene (Korsak et al., 1992[52]). Korsak et al. (1994[54]) reported an 18.5 % fall in erythrocyte, 35 % increase in leukocytes, and a significant decrease in hemoglobin in rats, which were intermittently subjected to 100 ppm of *m-*xylene. Jenkins et al. (1970[45]) observed that leukocyte counts were significantly increased in rats and dogs which were occasionally subjected to *o-*xylene for six weeks.

### Musculoskeletal effects

A study was conducted on workers exposed to xylene (14 ppm) indicated a reduction in grasping and muscular power in the extremities (Uchida et al., 1993[115]), although these special effects on muscles are not direct, but, elicited by involving the concerned neurons. However, human/animal data, about xylene exposure regarding the direct involvement of musculoskeletal system is not available.

### Gastrointestinal effects

Different studies have reported symptoms like nausea, vomiting, poor appetite and gastric discomfort of gastrointestinal tract (GIT) in workers exposed to xylene vapors (Klaucke et al., 1982[49]; Uchida et al., 1993[115]; Nersesian et al., 1985[72]). Although, above mentioned symptoms were subsiding after cessation of xylene contact. Exposure to xylene and some other unknown substance for about 2 weeks resulted in vomiting and anorexia in patients' admission in hospital later snuffling painting; although nausea was also observed in male acutely exposed for two hours (Sarmiento et al., 1989[93]; Ernstgård et al., 2002[29]). Goldie (1960[35]) reported that xylene exposure caused gastric discomfort and nausea.

Animal data about xylene exposure regarding the direct involvement of gastrointestinal system remain under study. There was severe congestion, pulmonary edema in lungs, no superficial erosions, and deep ulcerations detected during examination of the mucosa of the gastrointestinal tract of an individual who expired after ingestion of an unknown amount of xylene (Abu et al., 1986[1]).

### Cardiovascular effects

Regarding cardiovascular effects in one study, tachycardia was reported only in one case out of nine exposed individuals to xylene. Although tachycardia has no adverse effect on heart function, heartbeat, or blood pressure (BP) subjected to mixed xylene for short periods (Gamberale et al., 1978[34]), and 200 ppm *m*-xylene (Seppäläinen et al., 1989[103]; Ogata et al., 1970[76]). Likewise, two labors subjected to a probably too high xylene in a manufacturing coincidence had normal PB, heart, and pulse rate in the hospital. While long term exposure to xylene with other aromatic hydrocarbons workers complained of headache, eye irritation, heart palpitations, indigestion, thoracic pain, cases of flushing, and an irregular electrocardiogram (ECG) (Kilburn et al., 1985[48]; Hipolito, 1980[38]). Though, the toxic effects of other chemicals could not be underestimated. 

Respiratory paralysis, inflamed thickness of the coronary arteries, bradyrhythmia, and asystole were observed in rats exposed to 1,000 mg m^-3^ xylene for four weeks (Morvai et al., 1975[64], 1986[65]). Other changes like ventricular repolarization disturbance and occasional arrhythmias; were seen in rats inhaling unknown concentration. However, the unidentified mechanisms of toxicity were not described (Morvai et al., 1975[64], 1986[65]).

### Respiratory effects 

Exposure of volunteers to xylene for a short period under control conditions caused dysfunction of the pulmonary system, and irritation of respiratory mucosa. A study showed nose and throat irritation in human exposed for 3-5 minutes to mixed xylene (Nelson et al., 1943[71]). An autopsy done by Morley et al. (1970[63]) showed that an expected 10,000 ppm of xylene leads to severe lung edema and lung obstruction with hemorrhage in the alveoli's. Excessive or over exposure to xylene can lead to edema of the lungs, which is a threat to life due to accumulation of fluid in the lung. Workers subjected in the similar occasion revealed occasional diffuse opaqueness in radiograms and abnormal sound of lung. Different studies indicate that short term experience to mixed xylene or individual isomers have same effects which cause irritation of nose and throat (Nelson et al., 1943[71]; Klaucke et al., 1982[49]; Carpenter et al., 1975[15]). Worker subjected to heated xylene in the chemical industry experienced dyspnea and throat pain (Narvaez and Song, 2003[70]). Significant increase occurred in irritation of nose and esophagus in workers subjected to long term mixed xylene (Uchida et al., 1993[115]). Long term professional contact to mixed xylene to an unknown amount of fumes has been related with dyspnea, damage, pulmonary function, and rapid breathing (Roberts et al., 1988[88]; Hipolito, 1980[38]). Abu et al. (1986[1]) reported that on autopsy examination of a person who expired due to the ingestion xylene in large amount, pulmonary fluid and stenosis existed. Death occurred from central mediated respiratory depression.

Different studies reported that experience to mixed xylene or xylene isomers at different concentration produces a 50 % reduction in breathing rate in mice (De Ceaurriz et al., 1981[20]; Korsak et al., 1988[51], 1993[53]). Comparison of individual isomers of xylene effects on respiratory system showed that *o*-xylene have more prolonged effects while, after that *m*-xylene have more irritant effect on the respiratory rate (Korsak et al., 1990[50]). Carpenter et al. (1975[15]) described that rats died due to exposure mixed xylene for four hours and in autopsy, atelectasis, hemorrhages, and fluid in the lung were observed. Different experimental studies have indicated that mixed xylene or xylene isomers decreased lungs surfactant levels, and decreased pulmonary microsomal enzymes activities (Day et al., 1992[18]; Silverman and Schatz, 1991[104]; Elovaara et al., 1987[28]; Patel et al., 1978[78]; Elovaara et al., 1980[26]; Toftgård and Nilsen, 1982[113]). Elovaara et al. (1987[28]) reported that *m-*xylene at 70 ppm for 24 hours diminished p-450 and other enzymatic activities in the lungs of rats. While, reducing pulmonary microsomal activity by inhibition of some selective enzymes can lead to injury in the lung due to the production of metabolites of xylene like methyl benzaldehyde, which also lead to anorexia (Smith et al., 1982[105]; Patel et al., 1978[78]; Carlone and Fouts, 1974[14]). Different studies have shown the adverse respiratory effects of xylene detected in different laboratory animals following short term or intermediate through the respiratory system showed the same effects as detected in human which are irritation of the respiratory tract, difficult breathing, decrease breathing, pulmonary pinpoint hemorrhages, fluid in the lungs, and pulmonary cell damaged (Furnas and Hine, 1958[32]; Korsak et al., 1990[50]; De Ceaurriz et al., 1981[20]; Carpenter et al., 1975[15]). Single oral doses of mixed xylene at high concentration in mice lead to difficulty in breathing. While daily doses of mixed xylene in rats and mice by gavage at 2,000 mg kg^-1^ day^-1^ caused difficulty in breathing right after the dose administered (NTP, 1986[73]).

### Hepatic effects

Two painters and labors subjected to mixed xylene at different amount exhibited elevated levels of serum transaminases (Morley et al., 1970[63]; Klaucke et al., 1982[49]). Histopathological examination revealed minor liver toxicity, which involved increase of rough endoplasmic reticulum (RER), autophagous bodies, separation of ribosomes from the RER, dropped glycogen amount, deformed mitochondria, change in the propagation of hepatocellular nuclei (Ungváry, 1989[116]; Tatrai and Ungváry, 1979[110]). In the human primary enzyme is CYP2E1 which converts xylene to methylbenzylalcohols to form methyl hippuric acid (Tassaneeyakul et al., 1996[109]). This is then excreted through the urine. Occupational workers exposed through different routes to xylene, and other chemicals lead to high D-glucaric acid amount in the urine, which show increased hepatic enzymatic activities (Dolara et al., 1982[21]).

Animal studies using rats indicate that mixed xylene or their individual isomers mostly persuade hepatic cytochrome P-450 contents, and also elevate liver enzymes activities. It also improved comparative liver weight, microsomal proteins, P-450 amount, propagation of endoplasmic reticulum, different enzymatic activities, and diminished hexo-barbital during siesta time (Elovaara, 1982[26]; Patel et al., 1979[79]; Toftgård and Nilsen, 1982[113]; Rydzynski et al., 1992[91]; Selgrade et al., 1993[102]; Toftgård and Nilsen, 1981[114]; Ungváry, 1989[116]). Different enzymatic activities and enlarged liver were observed in a study in which rats were subjected to 4750 mg m^-3^ *o*-xylene for 1 year. Electron microscopic examination revealed negligible effect on mitochondria and the propagation of endoplasmic reticulum due to the huge number of peroxisomes (Tatrai et al., 1981[111]). Acute or intermediate ingestion of mixed xylene or their individual isomers caused elevation of cytochrome b5, hepatic weight, and hepatic enzyme (Pyykkö, 1980[82]; Condle et al., 1988[17]; Elovaara et al., 1989[27]; Ungváry et al., 1979[117]). Exposure to mixed xylene of rats for ninety (90) days leads to 13-27 % increase in weight of liver (Condle et al., 1988[17]). In the primary step of xylene break down, which occur in the liver oxidation of dimethyl groups attached on the side of xylene leads to methyl benzoic acid. In the second step, metabolites conjugate with glycine (Riihimäki et al., 1979[86]). When there is no glycine, another pathway started, in which xylene metabolites are conjugated with glucuronic acid to form xylenols. 

Generally, in animals' oral exposures to mixed xylene, moderate change in the liver is shown. Ultra-structural changes in liver enzymes actions indicate enlarged metabolic and biotransformation action, but there were no such changes on microscopic examination autopsy of liver (Ungváry, 1989[116]). While other studies have shown a rise in serum alanine aminotransferase post oral administration of *m-*xylene at 800 mg kg^-1^ daily (Korsak et al., 1994[54]; Elovaara et al., 1989[27]). 

### Renal effects

Various studies have found an increased acidity of kidney tubules (Sarmiento et al., 1989[93]), declined creatinine in the urine, and hematuria as a result of xylene exposure (Morley et al., 1970[63]). 

Kidney effects due to xylene were depended on concentration and dose which lead to aggregation of *m-*xylene in the fats of kidney in the periphery (Toftgård and Nilsen, 1982[113]; Elovaara, 1982[25]; Carpenter et al., 1975[15]). Other enzymatic activities and increased relative weight of the kidney were also detected in rats with different concentration of xylene (Wolfe, 1988[127]; Elovaara et al., 1989[27]). Histopathological evaluation revealed minimal chronic renal disease. Though, urine outcome was usual, the primary adverse effects detected was rising in a change in hyaline droplet in male rats and damage of kidney in the female rats which lead to cell damage (Condle et al., 1988[17]). 

### Endocrine effects 

In one study on dogs, mixed xylene at a concentration of 810 ppm created no noxious effects on the endocrine system (Carpenter et al., 1975[15]). The different studies which were done on developmental, reproductive, and long term exposure to xylene have not studied endocrine system toxicity. 

### Ocular effects

Studies reported that direct contact of eye to heated xylene can lead to hemorrhagic eye, conjunctiva, and intolerance to light, irritation and incomplete loss of epithelium. While in one case exposure loss of cornea, melting of substantia properia, and sub-conjunctival redness were observed. So it is concluded that chemical injury lead to enhance eye effects with thermal and physical injury, which finally lead to impaired vision or blindness (Narvaez and Song 2003[70]; Ansari, 1997[7]).

### Immunological and lymph reticular effects

Decreased lymphocytes and serum complement were detected in workers thoroughly subjected to xylene, benzene, and toluene; whereas there were no toxic effects on lymphocyte formation (Smolik et al., 1973[106]; Moszczynsky and Lisiewicz, 1983[68]). No conclusion can be made due to the available data regarding the xylene exposure and immunological effects due to workers simultaneous exposure to other industrial chemicals. The data revealed the harmful effects like reduction in weight of the spleen and thymus were observed in rats exposed to 2,000 mg kg^-1^ day^-1^ to xylene isomer for ten days (Condle et al., 1988[17]). There was no evidence of change accompanied by autopsy examination. 

### Neurological effects

Many experimental studies have revealed that xylene can cause nervous system abnormality in human. Xylene and their individual isomers lead to slow respond to external stimuli, alter memory, imbalance of body gait, and incoordination (Savolainen et al., 1979[97]; Dudek et al., 1990[22]; Savolainen and Riihimäki, 1981[96]; Savolainen et al., 1985[98]; Riihimäki and Savolainen, 1980[87]; Savolainen et al., 1984[95]).

Excitation and anesthesia were the well-defined neurological effects as a result of xylene exposure reported by Fang et al. (1996[30]). Moreover, Recchia et al. (1984[84]) reported xylene-induced coma which persisted for 26 hours in individuals exposed to high concentration of xylene. 

It has been observed that ingestion of 400 mg kg^-1^ of xylene lead to emotional exhaustion, incoordination, and bent posture of rats; reduce hind leg effort, difficult respiration and vibration (NTP, 1986[73]). Different other studies have reported the neurotoxic toxicity in rats comprising of spams, impulsiveness, hyper salivation, bleeding from the nose, and aggression after exposure to xylene (Wolfe, 1988[127]; Condle et al., 1988[17]). While some other important signs and symptoms in mice that had been seen are exhaustion, weakness, tremors, instability, and partial paralysis of the hind limbs (NTP, 1986[73]). But these studies have shown that these neurological effects are not only associated with xylene, but also the involvement of other chemicals cannot be excluded. Workers exposed to mixed solvents containing xylene for a long time developed slow down the nerve signaling specially in radius and tibia, while other symptoms were also observed like cramp, numbness, and weakness (Jovanoviæ et al., 2004[46]). Experimental studies showed that exposure via inhalation route to mixed xylene or their different isomers possess neurotoxic properties. The neurotoxic effects observed involved impulsiveness, cancer, altered vision, behavioral changes, incoordination, seizure, elevated respiration, hyperactive to stimuli, spams, and reduce acetylcholine (Molnar et al., 1985[62]; Korsak et al., 1988[51]; Andersson et al., 1981[6]; Honma et al., 1983[40]; Bushnell, 1988[13]; Savolainen et al., 1978[94]; Rank, 1985[83]). Alteration in neurotransmitters and decreased α-adrenergic binding in the hypothalamus were observed in animals (Rank, 1985[83]; Honma et al., 1983[40]). 

### Reproductive effects

Few epidemiological studies have reported reproductive toxicity of xylene. In China a study was conducted on workers who were exposed to mixed organic solvents in the petroleum industry. The result of this study showed that such a mixture of organic solvents caused an increase in the prevalence of oligomenorrhea (Cho et al., 2001[16]). There is another report on women exposed solvents containing organic aliphatic and aromatic hydrocarbons. And exposure to these solvents caused adverse outcome on reproductive hormones like reduction of pregnanediol 3-glucuronide (pd3G) in corpus luteum phase, pre-ovulatory luteinizing hormone (LH), and estrone 3-glucuronide, and higher follicle phase pd3G. Moreover, the contribution of xylene in the incidence of such effect was more than 50 % (Reutman et al., 2002[85]). Regarding male infertility, there are some reports of decrease spermatozoa viability, and decrease motility along with lower acrosin action release from spermatozoa which aid in penetration of the zona pellucida, decreased γ-Glutamyl transferase action, lactate dehydrogenase C4 (LDH-C4) and elevate the fructose level as a result of xylene exposure (Xiao et al., 2001[129]).

### Developmental effects

Individual isomers of xylene and mixed xylene cause toxic effects on the reproductive system and fetuses in laboratory animals. Studies have reported the subsequent effects in experimental animals like deformities in fetus skeletal, increased interruption in bone formation, redness and blood in fetus organs, and also lead to reduce fetus weight (Ungvary and Tatrai, 1985[118]; Ungvary, 1985[120]; Mirkova et al., 1982[61]; Balogh et al., 1982[12]; Hass and Jakobsen, 1993[36]). Marks et al. (1982[58]) reported that in mice orally exposed during gestation days 6-15 to mixed xylene lead to reduce fetal body weight and cleft palate. Significantly 3.5 % mortality occurred in mother at a concentration of 3,100 mg kg^-1^ day^-1^ to mixed xylene. Xylene leads to reduced fetus weight and specially develops offspring size with skeletal deformities. So it was concluded that xylene in huge amount lead to a fetus and parental toxic effects (Saillenfait et al., 2003[92]).

In one case control study, a case control study was done in which workers were exposed organic aromatic and aliphatic solvents; however, there was no evidence that abortion was due to xylene (Lindbohm et al., 1990[56]). Taskinen et al. (1994[108]) reported that there was spontaneous still birth or abortions among women who were working in laboratories subjected to xylene and formalin mostly. Mirkova et al. (1979[60]) have noticed that the enzyme responsible for acetylcholine hydrolysis cholinesterase oxidase was reduced in parent brain and the fetus. Xylene exposure through the skin could lead to a prominent change in fetus enzymes; while trailing by ingestion route lead to maternal death, delay development, and cleft palate. From this trail it is concluded that *p-*and *o-*xylene isomers seemed to be more toxic than *m-*xylene to progeny (Hood and Ottley, 1985[41]).

## Mechanisms of xylene toxicity

The lipid loving properties of xylene had made it easy to dissolve the lipid membrane, so it has the capacity to irritate dermis and mucous membrane of respiratory, eyes, and GIT system (Riihimäki et al., 1979[86]). This lipophilic property of xylene makes it able to induce anesthetic and narcotic properties, which is same for individual isomers as well (Fang et al., 1996[30]). The proper mechanism is not fully understood, however the chemical interaction with the cell membrane, which change the permeability and the nerve impulse signaling between nearby cells (Hood et al., 1985[41]; Tiihti, 1992[112]). The *p-*xylene isomer is responsible for provoking exaggerated reaction in laboratory animals (Fang et al., 1996[30]). Rats subjected to *m-*xylene at huge amount lead to 28 % rise in t-butyl bicycle-phosphorothionate, a molecule to cocculin/epileptic seizures, which bind with the GABA receptor were detected in the brain (Ito et al., 2002[44]). However, *m-*xylene has harmful effects on the coordination due to the rise in the inhibitory effects of GABA in the brain. 

In humans, CYP2E1 is the most important and rich enzyme in the liver, which break down the xylene to their corresponding metabolites, and finally form methyl hippuric acid (Tassaneeyakul et al., 1996[109]); while other enzymes lead to hydroxylation of xylene isomer forming 2,4 dimethyl phenol. Xylene is highly toxic to the liver and it will lead to apoptosis due to high production of caspase-3 and caspase-9 leading to apoptosis (Al-Ghamdi et al., 2003[5]; 2004[4]). The studies suggest that acute high level exposure to xylene lead to production of CYP2E1, which causes the assembly of oxidative intermediates and subsequent necrosis. The authors suggest that this mechanism would be related to acute high level exposure. Another study suggests that inhalation of* m-*xylene exposure for 6 hours lead to reduce in cytochromes like CYP 2B1, 2E1 and 4B1 in lung and 2B1 and 2E1 in the nostril mucosa (Vaidyanathan et al., 2003[121]).

**Figure 3:**
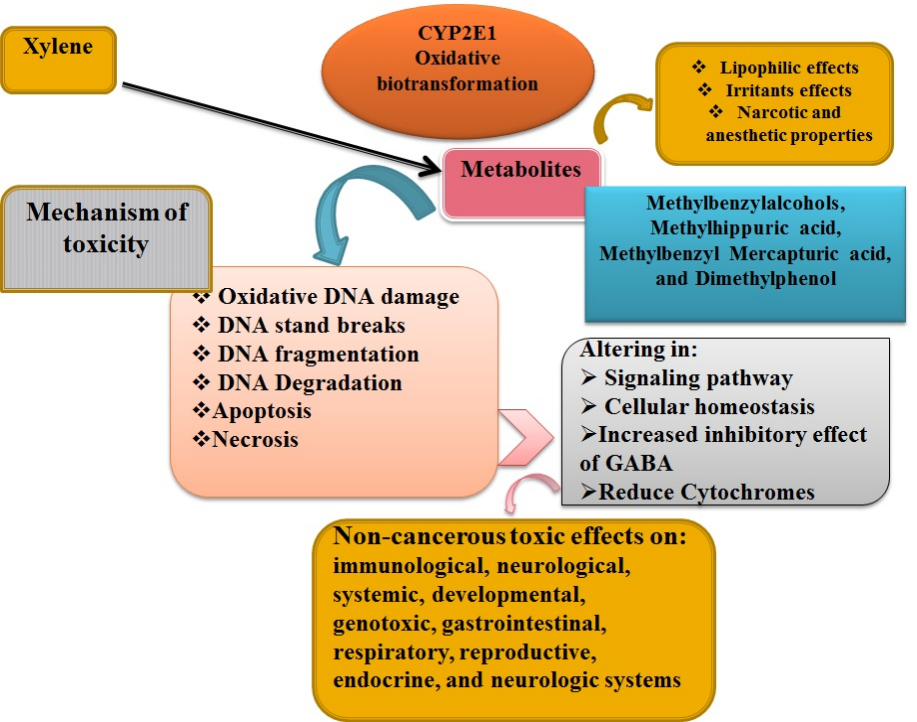
A schematic figure illustrating xylene metabolism, mechanism of toxicity and toxic health effects on biological systems

In short term bioassays from rats dermally subjected to xylene, it has not been shown that xylene cause mutagenic effects, but DNA fragmentation and disintegration was observed in the skin of rats (Rogers et al., 2001[89]). The whole summary of the mechanism of toxicity and toxic health effects is shown in Figure 3(Fig. 3). Dissemination of xylene to cell also alters cell membrane, which leads to DNA destruction and formation of nucleases from the membrane, which finally lead to death of cells due to direct and high exposure to xylene.

## Discussion

Xylene enter the body and cause toxic effects on immunological, gastrointestinal, respiratory, developmental, reproductive, and CNS as shown in Table 1(Tab. 1). 

References in Table 1: Carpenter et al. (1975[15]); Nelson et al. (1943[71]); Elovaara et al. (1987[28]); Patel et al. (1978[78]); Silverman and Schatz (1991[104]); Toftgård and Nilsen (1982[113]); Ungváry et al. (1980[119]); Andersson et al. (1981[6]); Korsak et al. (1993[53]); Molnar et al. (1985[62]); Padilla and Lyerly (1989[77]); Vodickova et al. (1995[122]); Balogh et al. (1982[12]); Saillenfait et al. (2003[92]); Honma et al. (1983[40]); Rank (1985)[83]; Rosengren et al. (1985[90]); Uchida et al. (1993[115]); Wolfe (1988[127]); Elovaara et al. (1989[27]); Condle et al. (1988[17]).

Also, short term inhalation of mixed xylene in humans results irritation of the throat, eye, and nose (Abu et al., 1986[1]; Ernstgård et al., 2002[29]; Korsak et al., 1988[51], 1992[52]; Uchida et al., 1993[115]).

Aromatic hydrocarbons are added to gasoline for sustaining high octane number and for most excellent anti-knock properties. Schauer et al. (2002[100]) have reported that *o-*xylene, and *m-/ p-*xylene released from non-catalyst-equipped gasoline-powered vehicles were 562 and 1,720 mg km^-1^, respectively; from catalyst-equipped gasoline-powered vehicle were 5.41 and 14.3 mg km^-1^, respectively; which show that petroleum contain xylene. Petrochemical additive such as xylene leads to pulmonitis. Currently, nearly all mixed xylene in minute quantity are added in petroleum for catalytic properties. Laboratory form of xylene constitutes of almost 15 % ethyl benzene, 20 % *o*-xylene, 44 % *m*-xylene, and 20 %* p*-xylene (Kandyala et al., 2010[47]). In the U.S. xylene concentration in indoor and outdoor air environment are ranging from 1-10 ppb and 1-30 ppb respectively. Though, 10,000 ppb concentrations of xylene in ground water have been reported (Fay et al., 1995[31]). However, these issues about developed and developing countries are growing. Therefore, the health hazards from xylene require additional attentions for management from side of environmental health organizations and agencies. Xylene not alone exists in the environment, but also with other organic solvents which is a silent threat to human health. It is hard to mention that in some developing countries, where xylene is inadequately monitored causes kidney, liver defects, cancer, nervous disorders, birth anomalies, circulatory problems, and human sterility (Askergren et al., 1981[9]; Astier, 1992[10]; Arp et al., 1983[8]; Carlone and Fouts, 1974[14]; Bushnell, 1988[13]).

Xylene individual isomers show the same effects on the liver, kidney, muscle, fat, and also on hemoglobin in the blood (Poulin and Krishnan, 1996[81], Adams et al., 2005[2]; Poulin and Krishnan, 1996[80]). The main mechanistic pathway responsible to convert xylene into their dominant metabolites methylhippuric acid is by hydroxylation (Ogata et al., 1970[76]). Different studies have reported that the adverse effects of each isomer of xylene are similar, like alveolar amount of xylene show effects of unconsciousness and ingestion of short term lead to decrease body weight gain (Fang et al., 1996[30]; Condle et al., 1988[17]). The mixed xylene had the similar outcome in developmental toxicity (Saillenfait et al., 2003[92]). However, if persons are not occupationally exposed to xylene, the huge amount can inhale from fire, smoking cigarette and other consumer products like paints containing xylene (Wallace et al., 1988[124], 1991[123]). People living near industries and chemical plants are at high risk, where xylene is not properly monitored. While some other studies indicated that xylene isomers lead to different toxicity, so no such effects illustrate that particular isomer is responsible for potent effects. *O-*xylene mostly shows effects on motor nerve coordination and impairs behavioral changes in rats (Korsak et al., 1990[50]; Moser et al., 1985[66]).

Currently, concentration of xylene is not constant in the environment, but a study was conducted which show that in the city areas of U.S. the xylene amount was 1-80 ppm in an environment (Merian and Zander, 1982[59]). Therefore, it means in villages or rural areas the concentration is expected to be lower. But the xylene usage and discharge patterns have changed greatly since these measurements, however, these above values are not constant and depend on each country regulation authority. And the exposure of xylene to general populations would be predictable due to use of products like pesticides, fuels, paint thinners, and varnishes (Fay et al., 1995[31]). However, in California the usages of xylene have been reduced due to replacement of oil base paints and varnishes, while the U.S. replaces aromatic hydrocarbons (toluene/benzene/ethyl benzene/xylene) in gasoline with methyl tart-butyl ether, which have considerably decreased population exposure. 

To help the public living or working near hazardous waste sites need to familiar with health hazardous effects of xylene. Taking into consideration the occupational, epidemiological, and experimental evidences brought in this review. Further, experimental research is still needed in regions with high exposure to xylene like the U.S., and histological laboratories in order to collect more evidences about the health hazards of xylene. More experimental studies should be carried out to help in understanding the toxicity and molecular mechanism of xylene and especially relevant with endocrine effects on diabetes.

## Conclusion

The purpose of the present study was to accumulate epidemiological and experimental data on xylene, which have non-cancerous health effects while high amount and duration can lead to blood toxicity. This study suggests implementing environmental monitoring systems and giving special attention in order to reduce the hazards of xylene. For this reason, all responsible bodies and researchers integrated research in those areas of high risk of xylene. Health and environmental pollution follow-up system should be developed. International and National health policies must be developed, for reduction of exposure of xylene. As mentioned that xylene is carcinogenic for blood components with other aromatic hydrocarbons, so single xylene should be evaluated both in occupational and experimental studies, their mechanism of toxicity in relation with general human health problems especially endocrine disturbances with particular reference to diabetes. So finally it is concluded that xylene emission is high in developed, developing as well as in underdeveloped countries due to extensive usage of petroleum refineries, vehicles, industries and chemical plants. So toxicologists give attention to its noxious effects with its long term use on public health and high exposure of xylene. Efforts to diminish the health hazards in the environment should be made to generate a safer living atmosphere by making the environmental health departments more familiar with the health hazards of xylene, safety measures and emergency procedures. From most of the sites xylene have no evidences of excess risk, however, some limited evidences of increased risk were found in association with other chemicals and singly. The adverse effects of xylene are well documented and their associations with other solvent are also evaluated. Usage of proper personal protective equipment is essential such as decent fume hood, proper white coat, and eye protector, mask to cover nose and mouth, while dealing with health hazardous chemicals like xylene. Acute and chronic xylene exposure should be an issue of concern for the population of all countries with special focus on those regions where xylene is observed in high concentration. 

## Acknowledgement

Authors wish to thank assistance of the Iran National Science Foundation.

## Conflict of interest

None to declare.

## Figures and Tables

**Table 1:**
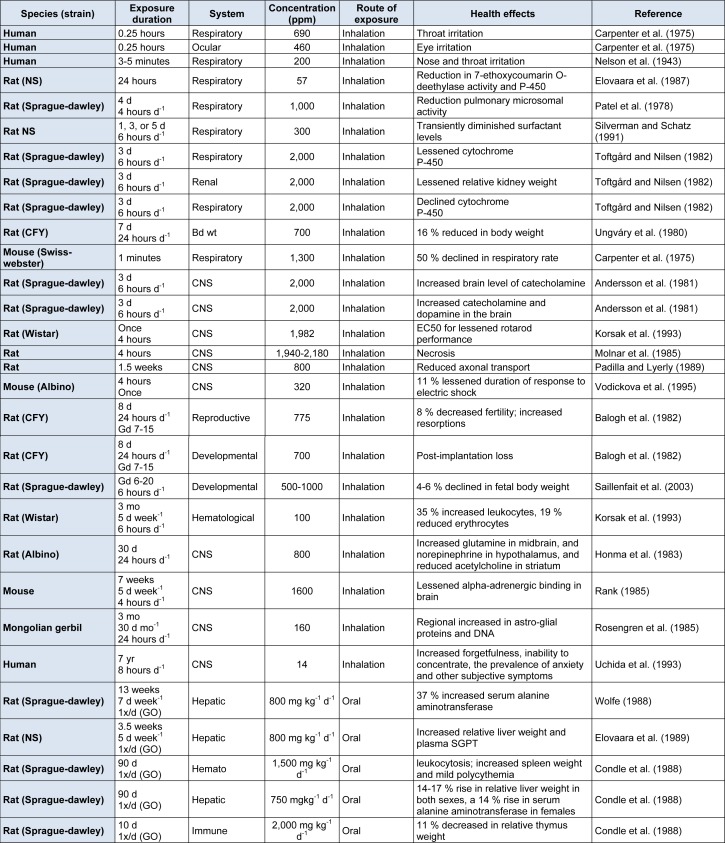
List of studies whose results show the association of xylene exposure and incidence of health effects on different systems

**Figure 1:**
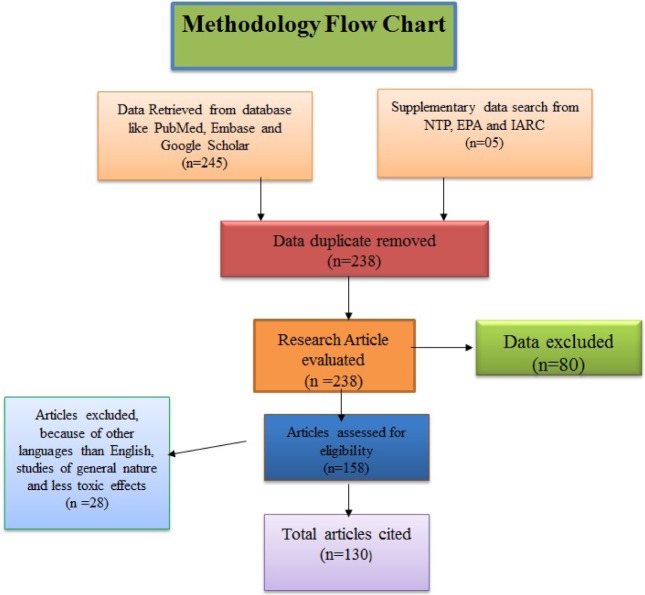
Flow diagram of included studies. The flow chart depicts the number of citation and resources materials that have been screened, excluded and/or included in the review.
